# Persistence of the uncanny valley: the influence of repeated interactions and a robot's attitude on its perception

**DOI:** 10.3389/fpsyg.2015.00883

**Published:** 2015-06-30

**Authors:** Jakub A. Złotowski, Hidenobu Sumioka, Shuichi Nishio, Dylan F. Glas, Christoph Bartneck, Hiroshi Ishiguro

**Affiliations:** ^1^Human Interface Technology Laboratory New Zealand, University of CanterburyChristchurch, New Zealand; ^2^Hiroshi Ishiguro Laboratory, Advanced Telecommunications Research Institute InternationalKyoto, Japan; ^3^Intelligent Robotics and Communication Laboratories, Advanced Telecommunications Research Institute InternationalKyoto, Japan; ^4^Department of System Innovation, Graduate School of Engineering Science, Osaka UniversityOsaka, Japan

**Keywords:** uncanny valley, anthropomorphism, human-robot interaction, multiple-interactions, eeriness, likeability, dehumanization

## Abstract

The uncanny valley theory proposed by Mori has been heavily investigated in the recent years by researchers from various fields. However, the videos and images used in these studies did not permit any human interaction with the uncanny objects. Therefore, in the field of human-robot interaction it is still unclear what, if any, impact an uncanny-looking robot will have in the context of an interaction. In this paper we describe an exploratory empirical study using a live interaction paradigm that involved repeated interactions with robots that differed in embodiment and their attitude toward a human. We found that both investigated components of the uncanniness (likeability and eeriness) can be affected by an interaction with a robot. Likeability of a robot was mainly affected by its attitude and this effect was especially prominent for a machine-like robot. On the other hand, merely repeating interactions was sufficient to reduce eeriness irrespective of a robot's embodiment. As a result we urge other researchers to investigate Mori's theory in studies that involve actual human-robot interaction in order to fully understand the changing nature of this phenomenon.

## 1. Introduction

The uncanny valley theory was originally presented by Mori (1970) in relation to a prosthetic arm. In the recent years it gathered a lot of attention in the fields of robotics, virtual agents, cognitive sciences, as well as in mass media. The uncanny valley hypothesis suggests a non-linear relationship between a robot's anthropomorphism and affinity. It proposes that by increasing humanlikeness of appearance of a robot we can also increase affinity with it. However, when a robot's appearance becomes a nearly perfect human representation, but is still distinguishable from it, people's emotional reaction instantly becomes strongly negative. Once the appearance of a robot becomes indistinguishable from a real human, the affinity with it reaches its optimum at the same level as for human beings. Furthermore, Mori suggested that movement of a prosthetic arm compared with a static arm will amplify the emotional response.

The uncanny valley is often used to explain people's rejection of anthropomorphic robots and virtual agents not only in science, but also in popular media as a reason for failure of computer-animated movies, such as The Polar Express. However, despite its wide adoption, there is relatively little empirical proof supporting it (Blow et al., 2006), e.g., the initial empirical work by Hanson (2006) and MacDorman (2006) indicated that humanlikeness might not be the only factor influencing perception of an object as eerie. Rendering style could be related with the uncanny valley for virtual agents (McDonnell et al., 2012). Moreover, it might be necessary to consider the effects of not only realism, but also the abnormality of artificial human appearance in order to investigate the uncanny valley phenomenon (Seyama and Nagayama, 2007; MacDorman et al., 2009). Mitchell et al. (2011) found that mismatch between appearance and voice can result in the uncanny valley. Furthermore, mismatch between appearance and movement of an android lead to stronger brain activation in the anterior portion of the intraparietal sulcus (Saygin et al., 2012), which could provide a neurological explanation of the uncanny valley. On the other hand, Piwek et al. (2014) reported that a realistic motion can improve acceptability especially of characters classified in the deepest point of the valley, which is against the original theory of Mori (1970) who suggested that motion will increase the uncanny effect. The uncanny valley was also reported for other primates. Monkeys looked longer at real faces and unrealistic synthetic faces than at realistic synthetic monkey faces (Steckenfinger and Ghazanfar, 2009).

### 1.1. Related work

Several potential explanations have been proposed for the uncanny valley. Apart from the neurological explanation (Saygin et al., 2012), other factors included empathy (MacDorman et al., 2013), perception of experience (Gray and Wegner, 2012), threat avoidance (Mori, 1970) or terror management (MacDorman and Ishiguro, 2006). Moore (2012) provided a mathematical model using a Bayesian model of categorical perception that can explain how stimuli containing conflicting cues can give rise to a perceptual tension at category boundaries that leads to the uncanny feeling. However, studies empirically investigating categorical boundary show that ambiguous morphs close to human endpoint induce positive affect rather than negative reaction suggested by the uncanny valley hypothesis (Looser and Wheatley, 2010; Cheetham et al., 2014). Furthermore, Poliakoff et al. (2013) found that for images of prosthetic hands intermediate humanlikeness was related with the highest eeriness, but within different categories of images increased humanlikeness was related with the lowest eeriness.

Vast research efforts are also dedicated to studying the dimensions of the uncanny valley. Especially, the term used originally in Japanese by Mori (1970)— *Shinwankan*—is particularly difficult to be translated to English. Various studies used different translations, such as familiarity (MacDorman, 2006), likeability (Bartneck et al., 2009a), affinity (Mori et al., 2012), eeriness (Ho and MacDorman, 2010) or empathy (Misselhorn, 2009), which might affect the comparability of the results. Moreover, also the humanlikeness axis of Mori's graph received empirical investigation (Cheetham et al., 2011).

The shape of the graph representing the uncanny valley was disputed. In one study toy robots and humanoids were preferred even over humans (Bartneck et al., 2007). The authors proposed that the relationship between humanlikeness and likeability resembles rather a cliff than a valley, where even perfectly realistic anthropomorphic robots are liked less than toy robots or mechanoids. These results imply that building highly humanlike androids might be unfruitful as their chances of acceptance are worse than for machine-like robots. In another study Bartneck et al. (2009a) found that a highly realistic robot (android) was liked as much as a human. Furthermore, they reported that an android's realistic motion did not decrease its likeability and questioned the existence of the uncanny valley. This result is in line with a study using virtual agents (Piwek et al., 2014). However, Ho and MacDorman (2010) pointed out that the scales used by Bartneck and colleagues were correlated with warmth and as a result with each other, which might have affected the results. Overall, the literature review shows lack of agreement between different studies regarding the dimensions and the shape of the uncanny valley, and indicates that Mori's theory could be too simplistic to accurately depict the relationship between human-likeness and perception of a robot or virtual agent. Moreover, it is not clear whether this theory has any actual consequences for interaction.

### 1.2. Does the uncanny valley affect human-robot interaction?

Despite being a common research theme, the effect of the uncanny valley hypothesis on Human-Robot Interaction (HRI) is unknown. Previous studies that investigated the uncanny valley used either images or videos of different targets that were supposed to induce the uncomfortable, eerie feeling (the exception is the work of Bartneck et al. 2009a that involved short-term HRI). However, these studies did not permit any interaction between participants and robots or virtual agents. In order to understand how the uncanny valley affects HRI, it is necessary to investigate it in studies that involve physically collocated robots as their physical presence can be an important mediating factor (Kiesler et al., 2008). Previous work suggests that people's attitudes toward robots change during interaction (Fussell et al., 2008), but it has never been empirically shown whether the uncanny feeling will persist.

Little is known about the lasting effect of the uncanny valley. It is implicitly assumed that this negative emotional response toward anthropomorphic technology will have enduring consequences and lead people to reject androids that are distinguishable from humans. Since this assumption has never been verified it is important to consider an alternative hypothesis in which the uncanny valley might lead to the negative emotional response only when the target is novel and the feeling of eeriness will disappear during the course of HRI. It is possible that the affective habituation caused by repeated interactions will allow people to get used to a machine that looks almost like a human, but still is not a perfect copy. Furthermore, the uncanny valley effect might decrease when an android interacts with a human in a friendly way. If that is the case, the effects of the uncanny valley on HRI might be limited to the pre-interaction phase.

### 1.3. Research questions

There is some empirical evidence suggesting only a short-term effect of the uncanny valley. In a study conducted during an ARS Electronica festival, visitors who had an opportunity to interact with an android and were interviewed afterwards, in majority, did not report an uncanny feeling (Becker-Asano et al., 2010; von der Pütten et al., 2011). Since this study had the form of an open interview that allowed people to talk freely about their experience, only a qualitative analysis was possible. Therefore, it is important to quantitatively show whether the uncanny feeling is experienced less during and after interaction with an android. Secondly, the analysis of the uncanny valley phenomenon with virtual agents indicates that there could be a relation between knowing an agent (previous exposure) and the uncanny discomfort experienced by people exposed to it (Dill et al., 2012). The decrease of previous exposure of an agent was related with higher discomfort.

Moreover, there are psychological theories that can suggest a relation between repeated exposures to a stimuli and the uncanny valley hypothesis: mere exposure effect and affective habituation. Zajonc (1968) showed that mere exposure to a neutral stimulus leads to increased positive affect toward it. On the other hand, for strongly positive or negative stimuli, the intensity of the reaction decreases after multiple exposures. This process is called affective habituation (Dijksterhuis and Smith, 2002).

The relationship between attraction and familiarity in interpersonal relations has been well documented. Positive relationships are a results of frequent face-to-face contacts (Ebbesen et al., 1976). However, if the person was disliked in the first place, greater familiarity will lead to greater dislike of that person (Ebbesen et al., 1976). This finding is consistent with work of Perlman and Oskamp (1971) who found that repeated exposure to unpleasant stimuli does not increase its likeability. Moreover, people rated more positively a person whom previously they have seen more frequently (Brockner and Swap, 1976) and they liked more others to whose ideas they were longer exposed (Brickman et al., 1975).

Four explanations have been proposed for the familiarity principle of attraction. Firstly, repeated exposure leads to increased processing fluency (Bornstein and D'Agostino, 1994), which on its own is affectively positive (Reber et al., 1998). Secondly, novel stimuli can produce uncertainty and negative reactions that diminish after a stimulus is found not to be harmful (Lee, 2001). Thirdly, due to classical conditioning, since most interactions are not aversive and rather mildly positive, others with whom people interact more often become paired with positive affect (Clark and Watson, 1988; Denrell, 2005). Fourthly, building on the previous explanation, repeated exposure creates an opportunity for interaction and these interactions are more likely to lead to rewarding social experiences (Denrell, 2005; Reis et al., 2011).

Mere exposure effect does not require interaction, but exposure is sufficient for it to occur and it has been reported for various types of stimuli (Bornstein, 1989). Although, Norton et al. (2007) proposed that in real interpersonal relations familiarity leads to dislike due to additional information about others making the less similar to oneself, Reis et al. (2011) using a live interaction paradigm showed that two previously unacquainted people shown positive affect with increased familiarity.

In relation with the uncanny valley, it is possible that for extreme stimuli the affective reaction will become weaker with people's increased familiarity with them due to affective habituation. However, for stimuli that were initially neutral, increased exposure could make them affectively more positive as a result of mere exposure effect.

This study is the first exploratory work that aims at investigating the effect of a robot's attitude and multiple interactions on the uncanny valley phenomenon by applying a live interaction paradigm in which actual HRI occurs. In particular, we focus on two aspects of interaction that could affect uncanniness of a robot: number of interactions and a robot's attitude toward a human. Moreover, we have chosen two of the most common components representing the y axis of the uncanny valley graph, *likeability* and *eeriness*, as they could be influenced differently by different aspects of HRI.

Likeability is an important factor affecting human-human relationships. Therefore, for long-term HRI it is expected to play an equally important role. There are multiple factors affecting human-human liking. One of the most important factors is history of interaction with a specific person. In particular we tend to like more others with whom we have positive rather than negative interactions (Smith and Mackie, 2007). Moreover, perception of a robot can be affected by its behavior (Goetz et al., 2003). Both positively and negatively behaving robots were anthropomorphized by people, but for an impolite behaving robot people had more mechanistic conceptions than for a positively behaving robot (Fussell et al., 2008). A robot that has a positive attitude toward a human could increase its likeability as would the classical conditioning explanation of mere exposure effect suggest. Similarly, an unfriendly robot could be liked less than it was before an interaction began. However, it is possible that an embodiment of a robot will play a role in affecting how strong effect its behavior will have on its likeability. Thus, we hypothesize that:

*H*_1*a*_: *A friendly behaving robot's likeability will increase with repeated interactions*.

*H*_1*b*_: *An unfriendly behaving robot's likeability will decrease with repeated interactions*.

On the other hand, we believe that previous exposure to a robot, irrespective of its behavior, will be more important for its perceived eeriness. Eerie robots could produce affective habituation and the initial strong negative emotional response will weaken with increased exposure. Similarly, for a robot that was initially perceived as neutral, repeated interactions can also positively increase the affective perception of it due to mere exposure effect.

In addition to looking at explicit measures, such as self-reports, we investigate implicit attitudes toward humanlike robots. Implicit measures assess automatic reactions and are not consciously controllable (De Houwer et al., 2009), and are incrementally valid (Steffens and Schulze König, 2006). In addition, implicit measures complement rather than replace explicit measures as they measure different aspects of the investigated attitude (Gawronski, 2002; Admoni and Scassellati, 2012). Therefore, we have also measured perceived eeriness of the robots implicitly. Thus, our next hypotheses are:

*H*_2*a*_: *Repeated interactions with a robot will reduce its explicit perceived eeriness*.

*H*_2*b*_: *Repeated interactions with a robot will reduce its implicit perceived eeriness*.

Recent work in HRI indicates that it might be necessary to consider anthropomorphism as a multidimensional rather than uni-dimensional phenomenon (Złotowski et al., 2014). These dimensions come from work on dehumanization—a process of depriving others of human qualities. Haslam (2006) proposed that there are two distinct senses of humanness: Human Uniqueness (HU) and Human Nature (HN). HU characteristics reflect socialization and distinguish humans from animals, e.g., intelligence, intentionality or secondary emotions. On the other hand, HN are inborn biological dispositions that distinguish humans from automata, e.g., warmth, sociability or primary emotions. Fussell et al. (2008) showed that anthropomorphism of a robot is not fixed and it changes during an interaction. It is currently unknown whether HU and HN dimensions of humanness attributed to a robot are also affected by the number of interactions or they are constant. In addition, previous work indicated that dimensions of mind attribution might be responsible for the uncanny valley phenomenon (Gray and Wegner, 2012). In particular, machines that are perceived as capable of experience, but not agency are also more uncanny. The dimensions of mind attribution and humanness are closely related (Haslam et al., 2012): agency reflects HU and experience reflects HN. Thus, our last hypotheses are:

*H*_3_: *HN, but not HU traits are related to a robot's perceived eeriness and likeability*.

## 2. Materials and methods

Our study was conducted using 2 × 2 × 3 mixed experimental design where a robot's embodiment (humanlike vs. machine-like) and attitude (positive vs. negative) were between-subjects factors, and number of interactions (Interaction I vs. Interaction II vs. Interaction III) was a within-subjects factor. We have explicitly measured a robot's perceived eeriness, anthropomorphism, likeability, and HN and HU dimensions of humanness. Furthermore, we used the Brief Implicit Association Test (BIAT) (Sriram and Greenwald, 2009) as an implicit measurement tool of eeriness. It is a computer-based program that requires participants to classify series of words into specified categories and measures the strength of the association between these concepts and attributes using participants reaction times.

### 2.1. Participants

Sixty native Japanese speakers were recruited by a recruitment agency for the study. The recruitment agency for part and full-time student jobs posted on its website a message informing about the possibility of participating in a study that involves a robot. Participants were paid ¥2000 for time compensation. All participants were undergraduate students of various universities and departments located in Kansai area. Only participants who previously participated in a study involving one of the robots where excluded from selection. Due to software failure, data of two participants was corrupted or not completely saved. Therefore, we had to exclude that data from the analysis. Out of the remaining 58 participants, 26 were female and 32 were male. Their age ranged from 18 to 36 years with a mean age of 21.47. The study took place at the premises of Advanced Telecommunications Research Institute International. Adequate ethical approval was obtained from the ATR Ethics Committee and informed consent forms were signed by the participants.

### 2.2. Materials and apparatus

**Figure 1:**
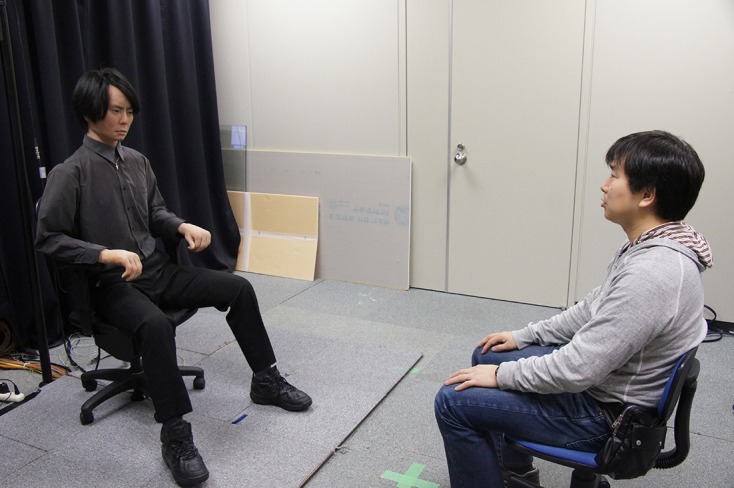
**Geminoid HI-2 and a participant**.

All the implicit and explicit measurements were conducted using PsychoPy v1.78 that was run on a laptop. Participants interacted either with Geminoid HI-2 or Robovie R2. Geminoid HI-2 is the second generation of androids built as a copy of a real human (see Figure 1). Geminoid is indistinguishable from a human being for several seconds, until people realize its slight imperfections that lead to a negative feeling (Ishiguro, 2006; der Pütten and Krämer, 2014). On the other hand, Robovie R2 is a machine like robot that has some human features, such as a head or hands. Therefore, Geminoid HI-2 represents a robot that is near the deepest point of the uncanny valley, while humanlike features of machine looking robot—Robovie R2—should make it highly likeable (Rosenthal-vonder der Pütten and Krämer, 2014). Furthermore, since the uncanny valley can be also caused by a mismatch between appearance and voice or movement (e.g., Mitchell et al., 2011; Saygin et al., 2012) in order to ensure that the Geminoid HI-2 will fall into the valley we have used a synthetic child-like voice and machine-like jerky movement that does not fit the appearance of a male adult. The same movements and voice were used for Robovie R2 where the mismatch does not occur. During HRI both robots expressed idle motion that was added to increase their animacy. Geminoid HI-2 showed movement resembling blinking and breathing, as well as idle movements of its hands and synchronization of its lips to its speech. As Robovie R2 does not have a mouth, identical idle behavior was not possible. Therefore, we implemented a slight head and hand motion during speech.

**Figure 2:**
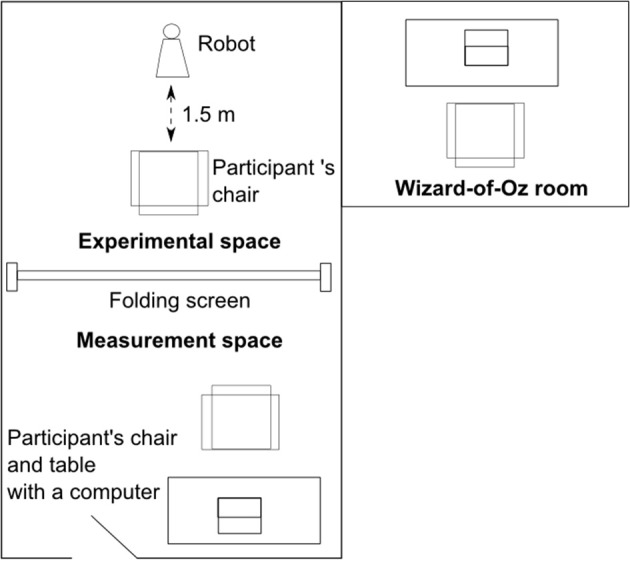
**Diagram of the experimental and measurement spaces and Wizard-of-Oz room**.

The experiment took place in a room that was divided into two parts that were separated by a folding screen in order to prevent seeing the other side (see Figure 2). In the experimental space a robot was placed and all HRIs occurred there. In the measurement space participants watched an introduction video that explained the order of the experiment, and they filled out all the questionnaires on a laptop. This ensured that participants did not need to judge the robot in its presence as that could have affected the results. The experimental space was equipped with cameras and the robot's behaviors were controlled by a Wizard-of-Oz who was sitting in another room.

### 2.3. Procedure

We used a live interaction paradigm. Participants were first shown an introduction video that explained the experimental procedure. They were told that the study involves creative and persuasive talking and they will need to convince a robot to give them a job based on the provided CV that was identical for all the participants. The experimenter ensured that participants understood the instructions and brought them to a computer. During all HRIs and filling out of questionnaires the experimenter left the participant alone in the room. The experiment was divided into 4 phases: pre-interaction video, Interaction I, Interaction II and Interaction III.

Although we have ensured that none of the participants previously interacted or participated in an experiment with the specific robot to which they were assigned, it was still possible that they have seen the robot elsewhere. In particular, in Japan it is common to see robots used in this experiment in various TV programs. Therefore, in order to minimize the differences in potential prior exposure in the pre-interaction video phase participants were asked to watch a short video (~15 s) in which a robot (either Robovie R2 or Geminoid HI-2) in few sentences introduced itself and its capabilities. The dialogue was identical for both robots. After the video participants performed the BIAT and filled out all the questionnaires.

**Figure 3:**
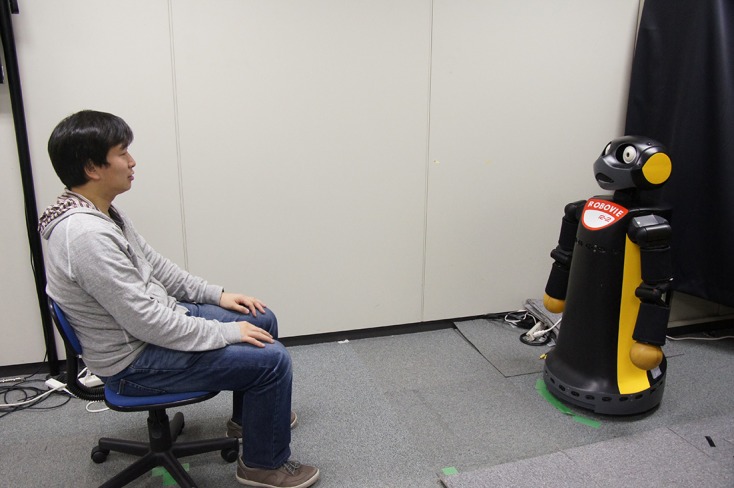
**Experimental setup**. Participant sitting in front of Robovie R2 during interaction.

During Interaction I, participants were taken to the experimental room and sat 1.5 m in front of a robot (see Figure 3). They were told to have a small conversation with it to become familiar before the actual job interview begins. The robot was introduced as *Robo*. During this conversation the robot asked participants 3 neutral questions (e.g., “Is it cold today?” or “Where did you come from?”). After a short conversation was finished participants were asked to fill out the same questionnaires as the first time.

In Interaction II, the experimenter provided a short job description for which the participant was instructed to apply. Participants were asked to apply for Engineer and Bank Manager positions. The order of interviews was counterbalanced between Interaction II and III. Furthermore, a participant received a CV of a person whom she was supposed to be imitating during the interview. The CVs were identical for all participants, but the gender of applicant was always the same as the real gender of a participant. Participants were asked to use it as a base of their responses, but they could invent the information required to answer the questions. In order to motivate participants for trying to perform the task as well as they can, they were informed by the experimenter that if they secure a job, they will be paid extra money as time compensation for their participation in the experiment. They were given 5 min to prepare for the interview. After that time elapsed, the experimenter collected the CVs and job description sheets, and brought the participant to the robot.

The interview began with the robot briefly describing the company and job position for which the participant was applying. After the introduction the participant was asked 3 job interview questions. The questions were generic and common for job interviews, e.g., “Please tell me about yourself?” or “What is your biggest weakness?” While the participant was responding the robot provided feedback using non-lexical conversation sounds and non-verbal communication. In the positive condition it either nodded or nodded and uttered “Un” (expression in Japanese of agreement with the speaker). In the negative condition it either shook its head or nodded its head and uttered “Asso” (expression in Japanese indicating lack of interest in what the speaker says that is rather rude). This feedback was initiated by the Wizard when it was appropriate for the natural flow of conversation, e.g., when a participant paused to think about her response.

After each question the robot thanked the participant and asked the next question. After the third question the robot informed the participant that it will announce later its decision whether to give a job to a participant (in fact the decision was never announced). Although the outcome was not provided directly to a participant, the announcement varied between the conditions. In the positive condition the robot hinted approval of what the participant said during the interview. In the negative condition it was not particularly pleased with a participant's responses suggesting them to consider applying elsewhere. At that point participants were asked to fill the questionnaires for the third time. This time multiple dummy questions regarding the interview were included. Interaction III was identical as Interaction II, but the CVs, job positions and questions asked by the robot were different. Participants were permitted to answer each of the questions freely and we did not measure the duration of interactions. The whole procedure took approximately 1 h.

### 2.4. Measurements

In the experiment we have used several questionnaires and the BIAT (Sriram and Greenwald, 2009) as dependent measures. We explicitly measured the robots' perceived eeriness and anthropomorphism on 5-point Likert scales derived from Ho and MacDorman (2010). Moreover, likeability was measured using the corresponding Godspeed scale from Bartneck et al. (2009b) (range 1–5). In order to establish the relationship between multi-dimensional anthropomorphism and the uncanny valley we have measured 2 dimensions of anthropomorphism: HN and HU on scales developed by Haslam et al. (2009). Both dimensions had 10 items and were measured on a scale from 1 (not at all) to 7 (very much) (e.g., “The *Robo* is… shallow”). This experiment is part of a bigger study that involved additional self-report scales that were collected at the same time and are not reported here. We used a validated version of likeability scale in Japanese. Perceived eeriness, anthropomorphism, HN and HU were available only in English. Therefore, we conducted a back-translation process to obtain their Japanese versions. We calculated reliability of each scale separately for each interaction round using Cronbach's α. According to Nunnally (1978) Cronbach's α > 0.6 is acceptable for newly developed scales for research purposes. Based on this threshold, all the scales, apart from HU were adequately reliable. The lowest Cronbach's α values during any of the three measurements were as follows: likeability α = 0.83, perceived eeriness α = 0.62, anthropomorphism α = 0.88, HN α = 0.65 and HU α = 0.54. Low reliability of HU scale indicates that the results for this scale should be interpreted with great caution.

Furthermore, we used BIAT (Sriram and Greenwald, 2009) as a computer-based implicit measurement tool of eeriness. BIATs involve participants classifying series of words into superordinate categories. The task involved combining concept classification (*“Robo”* vs. “Human”) with an attribute classification (“Eeriness” vs. “Non-eeriness”). We were interested in measuring the strength of association between *“Robo”* and “Eeriness.”

In the BIAT only 2 categories are displayed on the screen at the time and in total 3 categories are being evaluated (“Interview Robot *Robo*,” “Human” and “Eeriness”). The fourth category (“Non-eeriness”) is called non-focal and was used only as a distractor (attribute word that does not belong to the categories that are being evaluated in a specific block) for “Eeriness.” The other 2 categories (“Interview Robot *Robo*” and “Human”) were used as distractors for each other. There were 2 blocks with 16 trials each that were repeated 4 times. The following stimuli were used: “Interview Robot *Robo*” (Automaton, Machine, Robot, Artificial), “Human” (Person, Natural, Mankind, Real), “Eeriness” (Eerie, Freaky, Spine-tingling, Shocking) and “Non-eeriness” (Reassuring, Numbing, Uninspiring, Boring).

**Figure 4:**
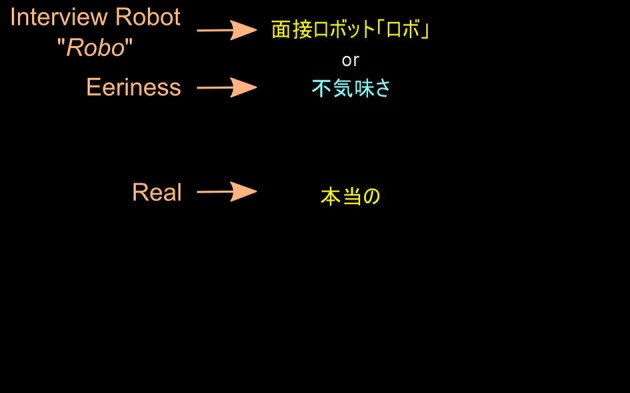
**A screenshot from the BIAT with English annotations**. Two classification concepts (“Interview Robot *Robo*” and “Eeriness”) and an attribute word (“Real”) are being classified by a participant.

At the beginning of BIAT, participants are presented with two categories that are being evaluated at the time (e.g., “Interview Robot *Robo*” and “Eeriness”) and the words that belong to each of these categories. During the actual classification task these categories are displayed in the top part of the screen. At the center of the screen appear series of words that either belong to these categories or not (see Figure 4). Participants are asked to press as fast as possible a “K” key if the word belongs to either of the categories or “D” key if it belonged to neither category. As an example, if the categories were “Human” and “Eeriness,” a participant should press “K” key if the target word is “Mankind” or “Freaky,” but “D” key if the word is “Artificial” or “Reassuring.” If a participant misclassified a word a red cross appeared on the screen. It remained there until the correct key was pressed.

Total time from the word appearing until the correct answer was provided was calculated with millisecond precision and was used to establish the strength of association between the categories. The idea of this task is that when an association between two categories is stronger, participants should be able to make their choices faster than for a pair of categories that are implicitly not associated with each other. The order of the BIATs was randomized and the order to blocks was counterbalanced.

## 3. Results

In the first step of the analyses we looked at the explicit and implicit measures. We then looked at the relationship between these different dependent measures. To analyze the data we conducted a series of Three-Way ANOVAs with embodiment and attitude as between-subjects factors, and number of interactions as a within-subjects factor. The assumptions of used statistical tests were met, unless otherwise specified.

### 3.1. Likeability

**Figure 5:**
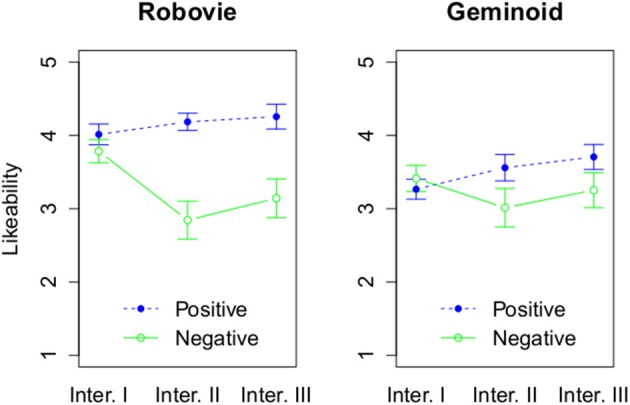
**The effect of 3 factors on likeability**. The rating of likeability based on attitude and interaction round, and grouped by a robot type.

First, we looked at the likeability and in particular how a robot's attitude can affect it in HRI. Due to violation of the assumption of normal distribution for parametric testing for anthropomorphism, we used a permutation test with 3 factors using the function aovp with 1000 iterations from the lmPerm package (Wheeler, 2010) using R (R Core Team, 2014). Likeability was significantly affected by the robots' attitude, *p* = 0.001 (see Figure 5). Positively behaving robots (*M* = 3.82, *SD* = 0.67) were liked more than negatively behaving robots (*M* = 3.24, *SD* = 0.9). Moreover, we found a statistically significant effect of embodiment with probability *p* = 0.01. Robovie R2 (*M* = 3.7, *SD* = 0.88) was liked more than Geminoid HI-2 (*M* = 3.37, *SD* = 0.78). In addition, we found a marginally significant interaction effect between embodiment and attitude, *p* = 0.07. Robovie R2 was more liked when it behaved positively (*M* = 4.15, *SD* = 0.54) than negatively (*M* = 3.26, *SD* = 0.94), *p* < 0.001. On the other hand, the attitude of Geminoid HI-2 did not significantly affect its perceived likeability.

Furthermore, we found a statistically significant interaction effect between robots' attitude and number of interactions, *p* < 0.001. During Interaction I, a robot's attitude did not affect its likeability. However, during Interaction II a robot's positive (*M* = 3.86, *SD* = 0.66) attitude increased its likeability compared to the negative attitude (*M* = 2.93, *SD* = 0.98), *p* < 0.001. Similarly, during Interaction III a robot's positive attitude (*M* = 3.97, *SD* = 0.69) resulted in higher likeability compared with a negatively behaving robot (*M* = 3.2, *SD* = 0.94), *p* < 0.001. The interaction effect between embodiment and measurement was also significant with *p* < 0.001. The difference was observed only during Interaction I when Robovie R2 (*M* = 3.9, *SD* = 0.56) was liked more than Geminoid HI-2 (*M* = 3.34, *SD* = 0.61).

### 3.2. Eeriness

**Figure 6:**
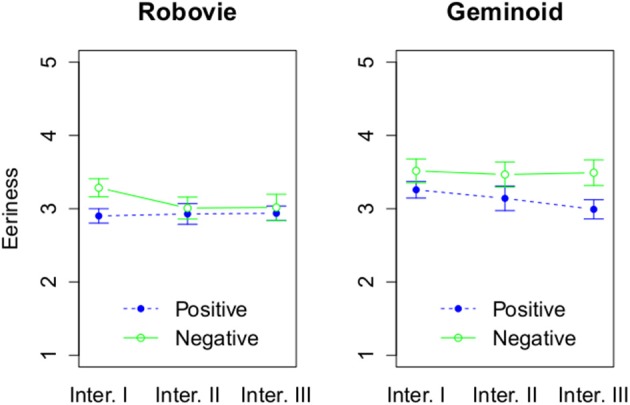
**The effect of 3 factors on explicit eeriness**. The rating of explicit eeriness based on attitude and interaction round, and grouped by a robot type.

The second component of the uncanny valley—eeriness—was measured explicitly and implicitly. We were interested in establishing the effect of repeated interactions on a robot's perceived eeriness. Explicit measure of eeriness showed the main effect of embodiment to be statistically significant, *F*_(1, 54)_ = 5.14, *p* = 0.03, η^2^_G_ = 0.07 (see Figure 6). Geminoid HI-2 (*M* = 3.31, *SD* = 0.62) was perceived as significantly more eerie than Robovie R2 (*M* = 3.01, *SD* = 0.51). Moreover, there was a significant main effect of attitude, *F*_(1, 54)_ = 4.27, *p* = 0.04, η^2^_G_ = 0.06. A robot behaving negatively (*M* = 3.3, *SD* = 0.64) was perceived as more eerie than when it behaved positively (*M* = 3.03, *SD* = 0.49). In addition, there was a main effect of number of interactions, *F*_(2, 108)_ = 3.1, *p* = 0.05, η^2^_G_ = 0.01. *Post-hoc* tests using the Bonferroni correction revealed that participants with marginal significance rated robots as more eerie after Interaction I (*M* = 3.25, *SD* = 0.52) than after Interaction III (*M* = 3.11, *SD* = 0.6), *p* = 0.08.

Apart from the explicit eeriness, we have also measured implicit eeriness. In the BIAT, the shorter the response time, the stronger the association between categories. The increased time would indicate that the association between a robot and eeriness is weaker. However, the reduced response time with increased number of interactions could be also due to participants improving at the task itself. Therefore, we have transformed the reaction times to z-scores within each interaction round, enabling the comparison of results between interactions. The conducted Three-Way ANOVA with embodiment and attitude as between-subjects factors, and number of interactions as a within-subjects factor did not indicate any statistically significant main or interaction effects.

### 3.3. Anthropomorphism

**Figure 7:**
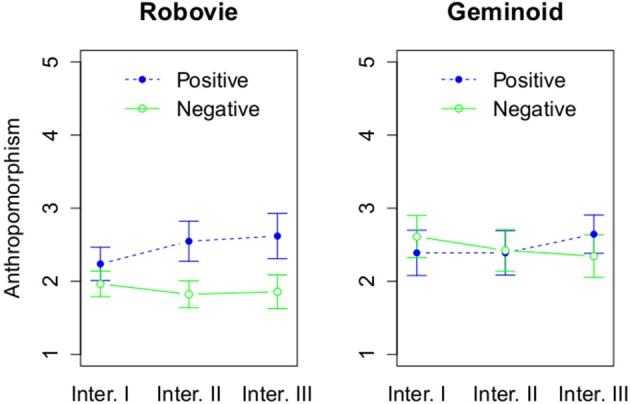
**The effect of 3 factors on anthropomorphism**. The rating of anthropomorphism based on attitude and interaction round, and grouped by a robot type.

We then looked at 1 and 2-dimensional measures of anthropomorphism. We expected that there would be a main effect of a robot's embodiment and in particular Geminoid HI-2 will be perceived as more humanlike than Robovie R2. Due to violation of the assumption of normal distribution for parametric testing for anthropomorphism, we used a permutation test with 3 factors using the function aovp with 1000 iterations from the lmPerm package (Wheeler, 2010) using R (R Core Team, 2014). We found a marginally statistically significant main effect of embodiment with probability *p* = 0.08 (see Figure 7). Geminoid HI-2 (*M* = 2.47, *SD* = 1.1) was more anthropomorphic than Robovie R2 (*M* = 2.17, *SD* = 0.92). Moreover, we found a significant interaction effect between robots' attitude and number of interactions with probability *p* < 0.001. Only during Interaction III a robot's positive attitude (*M* = 2.63, *SD* = 1.07) resulted in higher likeability compared with a negatively behaving robot (*M* = 2.11, *SD* = 1.02), *p* = 0.05.

**Figure 8:**
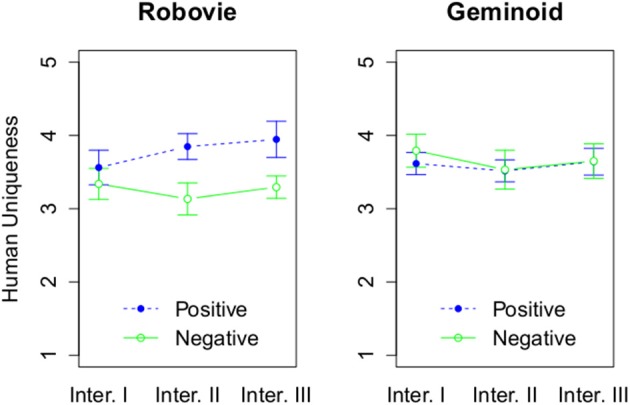
**The effect of 3 factors on Human Uniqueness**. The rating of Human Uniqueness based on attitude and interaction round, and grouped by a robot type.

We then proceeded to the 2-dimensional measurement of anthropomorphism to investigate its relation with the uncanny valley. The results related to the model of anthropomorphism proposed by Złotowski et al. (2014) will be discussed in another paper. In line with previous research, we did not find statistically significant main or interaction effects for the HU dimension (see Figure 8).

**Figure 9:**
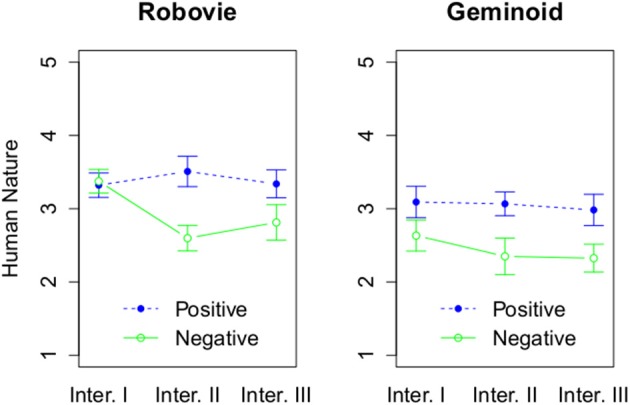
**The effect of 3 factors on Human Nature**. The rating of Human Nature based on attitude and interaction round, and grouped by a robot type.

On the other hand, we found a main effect of embodiment, *F*_(1, 54)_ = 5.13, *p* = 0.03, η^2^_G_ = 0.07 on HN dimension (see Figure 9). Robovie R2 (*M* = 3.16, *SD* = 0.77) was attributed more HN traits than Geminoid HI-2 (*M* = 2.74, *SD* = 0.85). In addition, there was a significant main effect of attitude, *F*_(1, 54)_ = 8.46, *p* = 0.005, η^2^_G_ = 0.12. Robots with positive attitude (*M* = 3.21, *SD* = 0.74) were attributed more HN than with the negative attitude (*M* = 2.67, *SD* = 0.85). There was also a significant main effect of number of interactions, *F*_(2, 108)_ = 7.39, *p* = 0.001, η^2^_G_ = 0.02. *Post-hoc* tests using the Bonferroni correction for the family wise error revealed that the robots were attributed more HN traits after Interaction I (*M* = 3.4, *SD* = 0.77) than after Interaction II (*M* = 2.88, *SD* = 0.87), *p* = 0.02, or III (*M* = 2.86, *SD* = 0.86), *p* = 0.02. Furthermore, there was a significant interaction effect between attitude and number of interactions, *F*_(2, 108)_ = 9.8, *p* < 0.001, η^2^_G_ = 0.03. Only for Interaction II [*F*_(1, 56)_ = 15.82, *p* < 0.001, η^2^_G_ = 0.22] and III [*F*_(1, 56)_ = 7.75, *p* = 0.007, η^2^_G_ = 0.12] the attitude had a significant effect.

### 3.4. Relationship between the uncanny valley and HRI factors

In the next step we looked at the relationship between different dependent variables used in this study in order to establish how the uncanny valley is related to factors that are important for HRI. We have calculated correlations between likeability, eeriness, 1 and 2-dimensional anthropomorphism, see Table 1.

**Table 1:** **Correlations between dependent measures using Pearson's *r* coefficient**.

	**Likeability**	**Eeriness**	**Anthropomorphism**	**HU**	**HN**
Likeability		−0.13	0.43^*^	0.33^*^	0.54^*^
Eeriness	−0.13		0.07	0.18	0.13
Anthropomorphism	0.43^*^	0.07		0.16	0.39^*^
HU	0.33^*^	0.18	0.16		0.36^*^
HN	0.54^*^	0.13	0.39^*^	0.36^*^	

* *p < 0.001*.

The following convention was used to determine the effect size of Pearson's r coefficient: small (0.1 ≤|r| <0.3), medium (0.3 ≤|r| <0.5), large (0.5 ≤|r|). There was a correlation with large effect size between likeability and HN, *r*_(56)_ = 0.54, *p* < 0.001. Furthermore, likeability had a medium effect size correlation with anthropomorphism [*r*_(56)_ = 0.43, *p* < 0.001] and HU [*r*_(56)_ = 0.33, *p* < 0.001]. Eeriness and likeability were not correlated.

## 4. Discussion

In this study we investigated the effect of repeated interactions and a robot's attitude on the uncanny valley phenomenon using a live interaction paradigm. In particular, we investigated the impact of these factors on a robot's likeability, as well as explicit and implicit measures of perceived eeriness. Explicit eeriness and likeability were not significantly correlated, which indicates that they measure different aspects of the uncanny valley. Although that might initially seem like an unexpected and counterintuitive finding, there are examples which show that negative correlation between eeriness and likeability is not necessary. People can dislike other people, but at the same time do not perceive them as eerie. However, there are also cases when eeriness is desirable, e.g., people who like to watch horror movies that might involve eerie creatures. Therefore, measuring both of the aspects can result in a richer picture than if we consider only one of them.

The analysis of likeability showed the more machine-like robot (Robovie R2) to be more liked than the highly humanlike Geminoid HI-2. Moreover, a robot's attitude toward a human interaction partner could be used to affect its likeability, with friendly robots being liked more than unfriendly behaving robots. However, the effect of a robot's attitude is not independent of its embodiment. The interaction effect between embodiment and attitude is especially profound in the case of a more machine-like robot. Although Robovie R2's positive behavior resulted in a small increase of likeability, it is the negative attitude that resulted in a drop of likeability ending at the level similar to the one observed for the negatively behaving Geminoid HI-2. In case of the latter robot, its attitude did not affect significantly its likeability. Thus, *H*_1*a*_ and *H*_1*b*_ are not supported.

**Figure 10:**
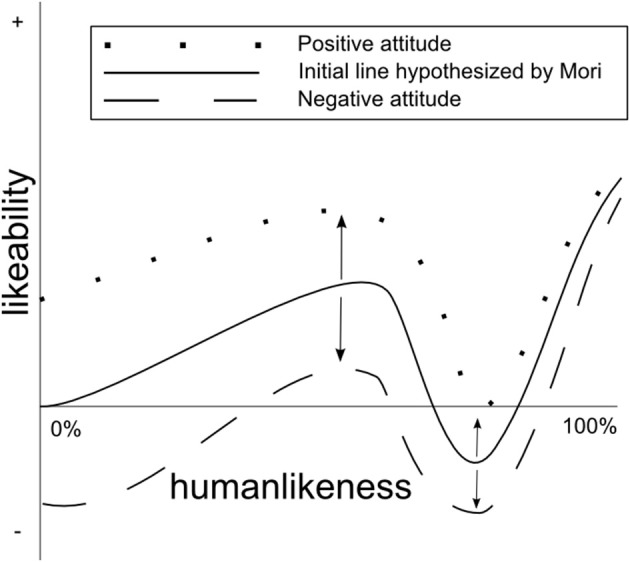
**Hypothesized effect of robots attitude on the uncanny valley**. Likeability of a robot will increase with its positive attitude toward a human interaction partner or decrease with its negative attitude. The effect will be stronger the less humanlike a robot is.

These results seem to indicate that a robot that is perceived as uncanny is not able to affect its likeability by a positive or negative interaction. In that sense its lower likeability is persistent. On the other hand, the impact of a machine-like robot's attitude is much greater and especially when it behaves negatively, it can lose all its initial likeability. The less humanlike a robot is, the stronger that effect could be. In this study we have used only 2 robots. In Figure 10 we present how hypothetically this relationship between humanlikeness and a robot's attitude on its likeability could look like for the broader spectrum of robots. Future, studies are needed in order to verify how well this figure represents robots with different levels of humanlikeness than those used in this study.

These findings on likeability can also provide a new perspective on the psychological theories related with the effect of familiarity. In particular, the results are consistent rather with mere exposure effect rather than affective habituation. As suggested by the work of Perlman and Oskamp (1971); Ebbesen et al. (1976), greater familiarity with an unpleasant stimuli did not enhance liking of Geminoid HI-2, which is in contradiction with affective habituation theory. However, in case of the more neutral stimuli (Robovie R2), its behavior during interactions affected its likeability. This supports the explanation of familiarity effect proposed by Denrell (2005); Reis et al. (2011) where repeated exposure creates opportunities for interaction and those interactions that are positive due to classical conditioning will lead to a favorable impression of a person, or in this case a robot. Therefore, in live HRI mere exposure to a robot is insufficient to induce a positive affect toward it and requires a positively toned interaction. However, in case of strongly unpleasant robot, even the positive behavior can be insufficient to enhance its liking.

**Figure 11:**
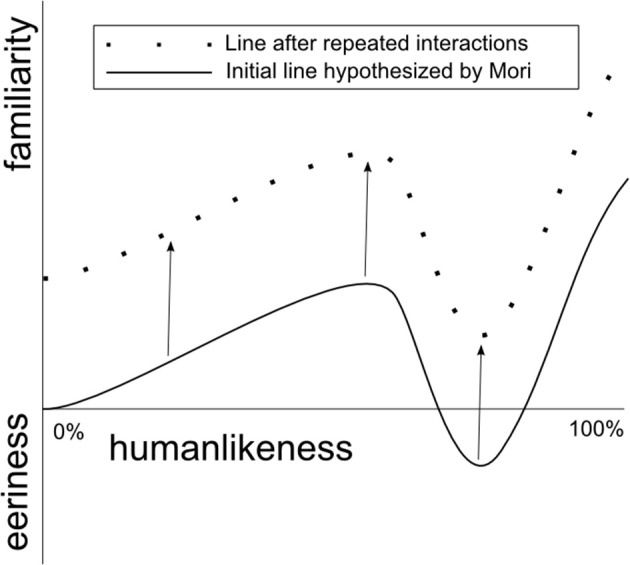
**Hypothesized effect of repeated HRIs on the uncanny valley**. The reduction of a robot's eeriness is relatively constant regardless of the level of its humanlikeness.

Looking at the second aspect of the uncanny valley investigated in this study— eeriness—we found that Geminoid HI-2 was rated as more eerie than Robovie R2. However, more interestingly we observed that after the last interaction both robots were perceived as less eerie than after interacting with them for the first time. This indicates that perceived eeriness is reduced with increased exposure to a robot. Moreover, this reduction is the same between robots that initially had different levels of eeriness, thus *H*_2*a*_ is supported. Therefore, although perceived eeriness of a highly anthropomorphic robot can decrease by merely increasing the number of HRIs, the gap between machine-like and humanlike robots remains relatively constant. This hypothesized relationship is presented visually in Figure 11. Future studies involving robots with different appearances are need to evaluate the graph's exact shape.

Since both robots were perceived as less eerie after multiple interactions, it is possible that both the mere exposure effect (Zajonc, 1968) and affective habituation (Dijksterhuis and Smith, 2002) were involved in this process. Geminoid HI-2, was initially perceived as an extremely eerie robot. In this case, it is possible that affective habituation process occurred and the affective reaction became weaker with increased exposure to it. On the other hand, for an initially neutrally looking robot (Robovie R2), additional exposures were sufficient to decrease its eeriness irrespective of its behavior. Therefore, the effect of familiarity on the perceived eeriness worked differently than for likeability where a robot's positive behavior was necessary to lead to a favorable impression. If familiarity effect of attraction affects also perceived eeriness an explanation of it that requires the interaction to be positive is not supported. The more probable explanations of the obtained results for Robovie R2 are that a novel stimuli that initially fosters wary reactions after repeated interactions is found to be benign (Lee, 2001) or that additional exposures might increase a robot's processing fluency (Bornstein and D'Agostino, 1994) as its appearance becomes more familiar. Since increased processing fluency is affectively positive, it is possible that this processing affect is then transferred to the robot leading to decrease in perceived eeriness. Previous research using computer graphics investigated the relationship between the uncanny valley and these effects: exposure (Burleigh and Schoenherr, 2015), exposure and perceptual fluency (Cheetham et al., 2014), perceptual fluency (Yamada et al., 2013), and novelty and exposure effects (Cheetham et al., 2011). These experiments support our findings that repeated exposure modifies how we perceive and evaluate humanlike looking entities. Our study shows that this notion can be also applied for HRI.

These findings on both likeability and perceived eeriness are relevant for HRI designers. A robot can affect its likeability by its behavior. However, that effect is much stronger in case of a more machine-like robot. In particular, a machine-like robot can swiftly stop being liked despite its appearance as a result of its negative behavior. It is much harder to increase the likeability of a robot which initially falls into the uncanny valley, as a friendly attitude is not sufficient to change it.

On the other hand, people are able to quickly get used to an unfamiliar appearance of a robot. In our study three short interactions were sufficient to reduce its perceived eeriness. However, that reduction was not found to be stronger for a more anthropomorphic robot. Therefore, the relative difference in perceived eeriness between the robots remained at the same level. Nevertheless, in this study we have enhanced the eeriness of Geminoid HI-2 by creating a mismatch between its appearance, speech and movement. It is possible that if the only source of eeriness of the robot was its embodiment, the effect of multiple interactions with it would be more profound. It is also noteworthy that perceived eeriness of Geminoid HI-2 after Interaction III reached the level of Robovie R2 after Interaction I. Therefore, Geminoid HI-2 remained perceived as more eerie only because perceived eeriness of Robovie R2 also decreased. It is possible that with higher number of interactions, after a machine-like robot reaches the optimum of its familiarity, the same level can be reached by a highly humanlike robot, such as an android.

We have also found that a negatively behaving robot was rated as more eerie than a positively behaving robot. However, this finding could be explained as a result of the HRI context used in this experiment. In Japanese culture it is not typical for an interviewer to express lack of interest during a job interview in such an explicit and rude way as a robot did in this experiment. Therefore, such an attitude could have led a robot to be perceived as more eerie than when it behaved in a way that is common during human-human job interviews.

The analysis of implicit eeriness using BIAT did not show any significant differences, thus *H*_2*b*_ is not supported. Therefore, in the current form BIAT might not be optimally suited as a measurement tool of eeriness. We speculate that this result could be due to weak association between a robot's category (“Interview Robot *Robo*”) that was displayed on a screen and the specific robot with which the participants interacted. Since implicit attitudes tend to change slower than explicit attitudes it is possible that our manipulation was too weak for modifying that attitude toward a specific robot. As a result, participants might have responded to the robot's category as being merely a representation of robots in general rather than their specific robotic interaction partner. In future studies, it might be beneficial to use a picture of a robot instead of a name as a representation of its category.

In line with the previous research, the HU dimension of anthropomorphism was not significantly affected by the embodiment of a robot. Furthermore, attribution of HN traits was affected by the embodiment and therefore more relevant to the uncanny valley, thus *H*_3_ is supported. However, in contrast with the previous work (Gray and Wegner, 2012) it was the less uncanny robot (Robovie R2) that was attributed more HN. Despite this dimension having more impact on the uncanny valley, the relationship looks to be more complex than initially proposed. The biggest difference between the work of Gray and Wegner (2012) and ours are the robots used in the experiments. In the former experiment a single robot was used that either had the back of its head visible or it had a humanlike face cover. The HN dimension is closely related with emotions and a robot that had no face is not capable of expressing emotions with facial expressions. Therefore, it was attributed less capability of experiencing (HN). In our experiment the default and fixed appearance of Robovie R2's face could be perceived as a smile. However, Geminoid HI-2 has a highly humanlike face that suggests that it can exhibit facial expressions. As a result participants might have had higher expectations, but during the interactions the robot's facial expression remained the same and was rather stern. That might have been perceived as the robot's emotional coldness and led participants to attribute less HN to it. Nevertheless, more research is needed to establish the relationship between HN and the uncanny valley. Furthermore, considering inadequately low reliability of HU dimension it is necessary to interpret these results with special care. It is possible that HU dimension is a different construct in Japan than in Western cultures.

### 4.1. Limitations and future work

In our experiment we have used only 2 robots that differed in their level of anthropomorphism. An alternative explanation for the obtained results could be that it is a robot's friendliness in appearance that is more important for its likeability than humanlikeness. We cannot exclude a possibility that there are differences along some other dimensions reflected by appearance. It is possible that if we used different pair of robots the interaction between embodiment and attitude would be reversed. In particular, Geminoid HI-2 has a stern looking facial expression, while the design of Robovie R2 could be perceived as cute and friendly with its big, childlike head. The appearance of Robovie R2 could invoke expectations for it to behave positively, and the mismatch between these expectations and the actual behavior of the robot could result in a strong decrease of its likeability. If a more friendly looking android, e.g., Geminoid F, was used in the experiment instead of Geminoid HI-2, it is possible that we would have observed a similar pattern of reactions to its unfriendly behavior as for Robovie R2. However, a question remains open why the opposite trend was not observed in case of Geminoid HI-2's mismatched positive attitude. Therefore, future studies should also include qualitative data that could help to understand why people perceive robots as eerie or likeable. Moreover, there could be demographic factors, such as age, gender or educational background, that work as moderators. The role of these factors on the uncanny valley is still not well explored.

The scale used for measuring anthropomorphism (Ho and MacDorman, 2010) in experiments of the uncanny valley was developed in a study that involved only static images of robots. However, contrary to expectations Robovie R2 and Geminoid HI-2 only marginally differed on perceived humanlikeness. Since previous work indicates that androids are perceived as more humanlike than machine like robots (e.g., Ho and MacDorman, 2010), the small difference between these 2 robots in our study must be due to other factors than merely embodiment. In order to increase the uncanniness of Geminoid HI-2 we used voice and movement that does not match its embodiment. However, the humanlikeness scale can be also affected by this manipulation as its items do not apply only to the embodiment, e.g., items rated by the participants include “Artificial”–“Lifelike” or “Fake”–“Natural.” As a result our manipulation not only made Geminoid HI-2 more eerie, but also less humanlike than if only its embodiment was evaluated.

This finding also points out that a robot's behavior can be a more important factor of anthropomorphism than its embodiment. The potential solution could involve development of a new scale of anthropomorphism that is not affected by potential mismatch of a robot's embodiment and speech or movement. Alternatively, before investigating the uncanny valley in interaction it would be possible to first rate a robot's humanlikeness by presenting the static robot with no HRI.

Another limitation of this study is that participants were allowed to freely interact with a robot for as long as they wanted. Therefore, we did not consider the interaction duration in this study, but only the number of interactions. It is possible that participants who interacted with a positively-behaving robot were encouraged by its positive feedback to provide more detailed answers for their questions and as a result interacted longer with a robot. This extended interaction could have also increased familiarity of a robot and reduced its eeriness. It is also possible that the duration of interactions was insufficient to lead to the affective habituation effect of an uncanny robot. The perceived eeriness of both robots was reduced as a result of repeated interactions. However, it is still possible that after a higher number of interactions, the affective habituation effect would become stronger for the more eerie robot. A long-term study with highly anthropomorphic robots could answer this question. In particular future experiments could involve longer interactions with a robot with sessions spread over multiple days.

Future work should also consider the dynamic nature of anthropomorphism. The complexity and multifaceted nature of anthropomorphism shows a potential challenge with investigating the uncanny valley in actual, long-term HRI rather than using images or videos that can focus only on a robot's embodiment. Previous work on the uncanny valley treated it as a static feature of a robot or virtual agent. However, Fussell et al. (2008) showed that a robot's anthropomorphism changes during HRI. The results of this study also point out that at least in case of Robovie R2, its attitude affected its perceived humanlikeness. Mori's hypothesis does not accommodate for such a finding. Studies of the uncanny valley should recognize that both anthropomorphism and uncanniness of a robot can be changing during HRI, and they should consider whether the uncanny valley should be investigated using the pre-interaction level of anthropomorphism based only on a robot's appearance or the level of anthropomorphism measured in HRI at the same point of time as measures of uncanniness.

This study was an exploratory work that for the first time investigated the uncanny valley in repeated HRIs. It shows potential benefits for researching the complexity of this phenomenon in studies that involve human interaction with a collocated robot. Nevertheless, at the same time, the obtained results indicate that if we want to understand the impact of the uncanny valley on HRI, future research must go beyond picture and video based studies and enable people to interact with robots. The great majority of studies have tried to find the origin of this phenomenon. This is a worthy goal. However, until we can show that Mori's theory has any significant (long-term) impact on HRI we risk spending resources on research that might be investigating an artificial problem. In the end, it matters very little whether a picture of a robot is perceived as eerie or disliked, if during an actual interaction with a robot, this effect will vanish as a result of behavior or interaction context factors being more prominent.

### Conflict of interest statement

The authors declare that the research was conducted in the absence of any commercial or financial relationships that could be construed as a potential conflict of interest.
